# Case Report: Epitheliotropic cutaneous presentation of multicentric diffuse large B-cell lymphoma in a dog

**DOI:** 10.3389/fvets.2026.1949754

**Published:** 2026-09-30

**Authors:** Gyu-Hyun Kim, Byung-Gee Ko, Se-Hoon Kim, Kyoung-Won Seo, Hwa-Young Youn, Min-Ok Ryu

**Affiliations:** Laboratory of Veterinary Internal Medicine, Department of Veterinary Clinical Science, College of Veterinary Medicine, Seoul National University, Seoul, Republic of Korea

**Keywords:** canine, case report, cutaneous involvement, diffuse large B-cell lymphoma, electrochemotherapy, epitheliotropism

## Abstract

Diffuse large B-cell lymphoma (DLBCL) is the most common histological subtype of canine multicentric lymphoma, whereas epitheliotropism is classically associated with cutaneous T-cell lymphoma. A 9-year-old neutered male Chihuahua was diagnosed with stage IVb multicentric lymphoma and achieved complete remission with a UW-25 L-CHOP protocol. However, an upper lip mass, cytologically consistent with lymphoma, developed during remission. Electrochemotherapy was performed for local control and resulted in marked reduction of the lesion. The dog later developed recurrent peripheral lymphadenopathy and multiple progressive cutaneous masses despite various chemotherapy protocols and was euthanized at week 59 because of progressive clinical deterioration. Histopathology confirmed diffuse large cell lymphoma in the submandibular lymph node, whereas the cutaneous lesions showed neoplastic lymphoid infiltration with multifocal epidermal invasion, mimicking epitheliotropic T-cell lymphoma. Immunohistochemistry (IHC) confirmed diffuse CD20 expression in both sites, with CD3 labeling limited to scattered background lymphocytes. These findings supported the interpretation of secondary cutaneous involvement of DLBCL with a highly unusual epitheliotropic pattern and emphasize the importance of IHC for accurate immunophenotypic classification of cutaneous lymphoma lesions.

## 1 Introduction

Lymphoma is a prevalent hematopoietic malignancy in dogs, with the multicentric form representing the most frequently encountered presentation (1). The World Health Organization classification system for non-Hodgkin's lymphoma is also applied in veterinary medicine, and diffuse large B-cell lymphoma (DLBCL) is the most common histological subtype (2). Affected dogs typically present with generalized peripheral lymphadenopathy, and extranodal involvement may occur in a subset of cases, including cutaneous involvement reported in approximately 10% of cases (2).

Cutaneous involvement by lymphoma may present with different histopathological patterns. Epitheliotropism is defined as the infiltration of neoplastic lymphocytes into the epidermis and adnexal structures (3). This pattern is characteristically associated with T-cell lymphoma, particularly in primary cutaneous forms (4–6). Retrospective studies of canine epitheliotropic lymphoma have demonstrated that the vast majority of cases exhibit a T-cell immunophenotype, with most expressing CD3 and lacking B-cell markers (7). In contrast, cutaneous B-cell lymphomas are uncommon in dogs and are generally classified as non-epitheliotropic lesions, which predominantly involve the deeper dermis (3). Epitheliotropic behavior in B-cell lymphoma is exceedingly rare even in human medicine (8, 9) and, to our knowledge, has not been documented in dogs.

Here, we describe a case of multicentric DLBCL in a dog that developed cutaneous involvement with an atypical epitheliotropic pattern. Immunohistochemistry (IHC) confirmed consistent CD20 expression in neoplastic cells from both the affected peripheral lymph node and the cutaneous lesions with epidermal infiltration. This report provides evidence that canine DLBCL may present with clinicopathological features distinct from those typically recognized in this disease.

## 2 Case description

### 2.1 Initial presentation and diagnosis

A 9-year-old neutered male Chihuahua dog was presented to the Veterinary Medical Teaching Hospital with a 5-day history of anorexia, lethargy, and enlargement of the bilateral submandibular lymph nodes. On physical examination, the dog had fever (39.2 °C), tachypnea (78 breaths/min), and generalized peripheral lymphadenopathy involving the bilateral submandibular, prescapular, inguinal, and popliteal lymph nodes. Blood analysis identified moderate regenerative anemia (hematocrit, 27.5%; reference interval [RI], 41–56.1%), elevation of ALT (154 U/L; RI, 5.8–83.3 U/L), AST (81 U/L; RI, 11.7–42.5 U/L) and C-reactive protein (81.2 mg/L; RI, 0–20 mg/L). Imaging findings included hepatosplenomegaly on abdominal radiographs, fine hepatic echotexture, and a heterogeneous “honeycomb-like” appearance of the splenic parenchyma on abdominal ultrasonography. Fine-needle aspiration (FNA) cytology of the enlarged lymph nodes, liver, and spleen indicated large cell lymphoma. No circulating neoplastic lymphocytes were identified on peripheral blood smear examination. Based on these findings, the dog was diagnosed with stage IVb multicentric lymphoma. Bone marrow aspiration and biopsy were not performed.

### 2.2 First-line chemotherapy and initial electrochemotherapy

The timeline of the patient's clinical course is summarized in Table 1. Treatment was initiated using a 25-week University of Wisconsin-Madison (UW-25) L-CHOP protocol with L-asparaginase (L-asp) induction (10). Complete remission was achieved during the first week of treatment and was maintained throughout the chemotherapy protocol. However, at week 25, a painful and erythematous mass measuring 2.34 × 2.77 × 0.9 cm was identified on the right upper lip, while no peripheral lymphadenopathy was detected (Figure 1A). FNA cytology of the lesion confirmed large cell lymphoma, which was considered an extranodal manifestation of the original disease.

**Table 1:** Clinical timeline of diagnosis, treatment, and disease progression.

Time	Major clinical event	Management and outcome
Week 0	Stage IVb multicentric lymphoma diagnosed	UW-25 L-CHOP initiated
Week 1	Complete remission achieved	Remission maintained during chemotherapy
Week 25	Solitary upper lip mass developed	First electrochemotherapy performed
Week 39	Generalized peripheral lymphadenopathy recurred	UW-25 L-CHOP reinitiated
Week 47	Multiple cutaneous lesions developed	Surgical excision and biopsy of selected lesions performed
Week 58	Progressive peripheral lymphadenopathy and cutaneous masses	Second electrochemotherapy performed
Week 59	Clinical deterioration	Euthanasia and postmortem examination performed

UW-25 L-CHOP; 25-week University of Wisconsin-Madison L-asparaginase-CHOP chemotherapy protocol.

**Figure 1:**
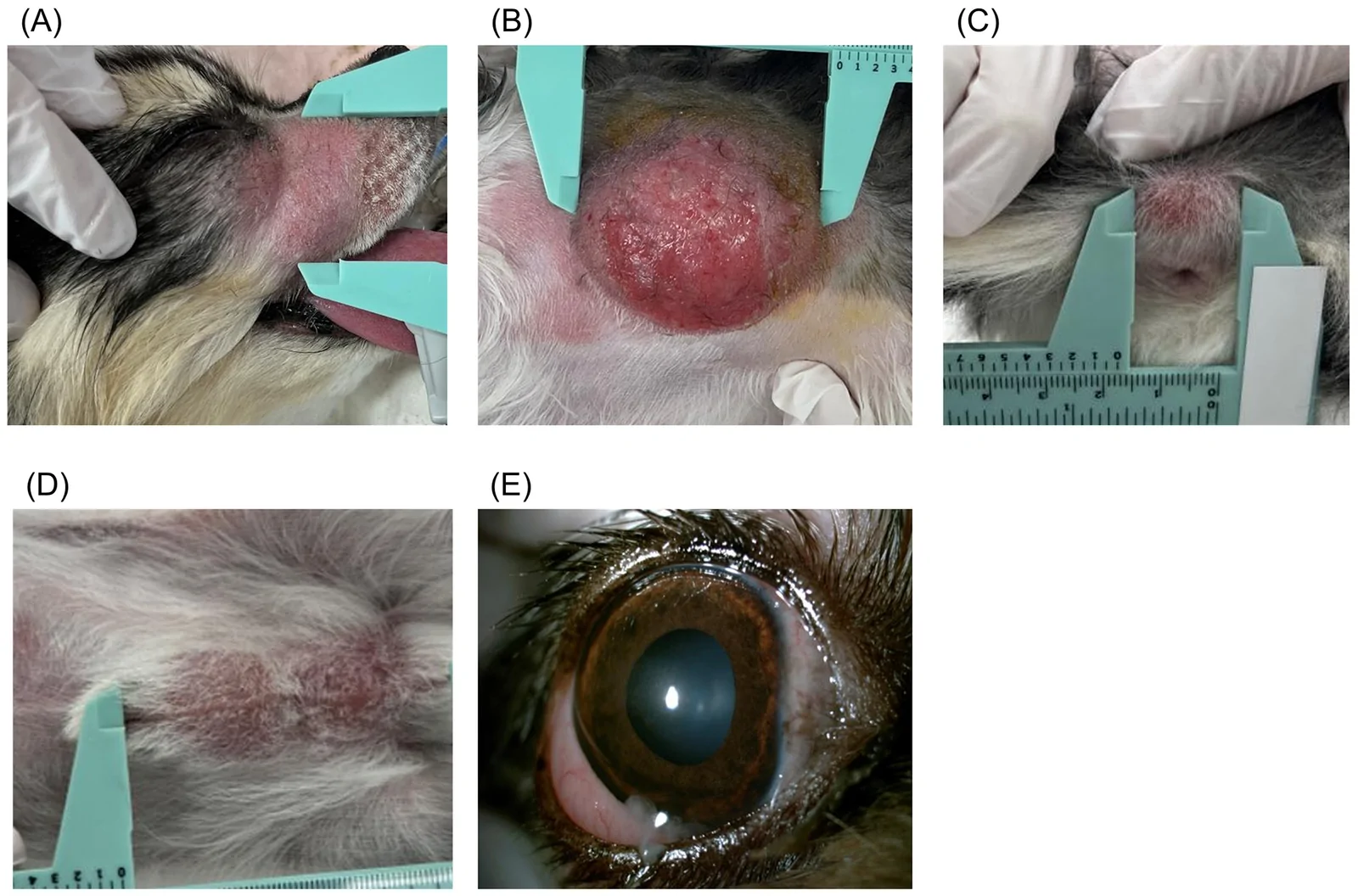
Gross appearance of multiple cutaneous lesions and conjunctival swelling. **(A)** A focal erythematous swelling is present on the right upper lip at week 25 before the first electrochemotherapy session. **(B–D)** Cutaneous lesions documented at week 47 before surgical excision and biopsy. **(B)** The left flank mass is alopecic and dome-shaped, with an erythematous and ulcerated surface. **(C)** The perineal lesion appears as a raised, erythematous nodule adjacent to the anus. **(D)** The inguinal lesion appears as an elongated erythematous plaque with mild surface crusting. **(E)** Focal swelling of the right lower conjunctiva is visible at week 48 as a pale pink, protruding lesion along the lower conjunctival margin.

Because no other lesions were detected on physical examination or abdominal ultrasonography, electrochemotherapy (ECT) was selected for local palliative treatment of the lip mass. ECT was performed using a BTX AgilePulse electroporation system (Harvard Bioscience, Holliston, MA). Bleomycin (15,000 IU/m^2^) was administered intravenously, followed by electroporation 8 min later. The electroporation protocol consisted of eight pulses with an amplitude of 600 V, a pulse duration of 50 μs, and an interpulse interval of 1 ms. After ECT, the lip mass decreased to a non-palpable size within 1 month.

### 2.3 Development and histopathological assessment of cutaneous lesions

Disease recurrence was noted at week 39, with generalized peripheral lymphadenopathy, enlargement of the lip mass, and development of a new cutaneous mass on the left flank. Cytologic evaluation of the lymph nodes and skin lesion supported lymphoma recurrence, and the UW-25 L-CHOP protocol was reinitiated. Although the enlarged lymph nodes resolved after administration of the L-asp and vincristine, the lip and flank masses rapidly recurred within one week, and additional cutaneous lesions developed despite ongoing chemotherapy. At week 47, multiple cutaneous masses were present on the right upper lip, left lower lip, left flank, ventral abdomen, left inguinal region, and right perineal region (Figures 1B–D). Among these, the left flank mass measured 6.9 × 5.6 × 5.0 cm and was associated with marked erythema, erosion, and pain. Due to its size, ECT could not be applied and palliative surgical excision was performed. In addition, incisional biopsy specimens were obtained from lesions on the ventral abdomen and perineal region.

All three cutaneous lesions showed similar histological features, characterized by dense dermal infiltration of intermediate to large neoplastic lymphocytes with focal invasion of the epidermis (Figure 2). This histological pattern mimicked epitheliotropic T-cell lymphoma, including mycosis fungoides. IHC was performed to determine the immunophenotype. The neoplastic lymphocytes were diffusely positive for CD20 whereas CD3-positive cells were limited to scattered small background lymphocytes. These findings confirmed DLBCL in all examined skin lesions.

**Figure 2:**
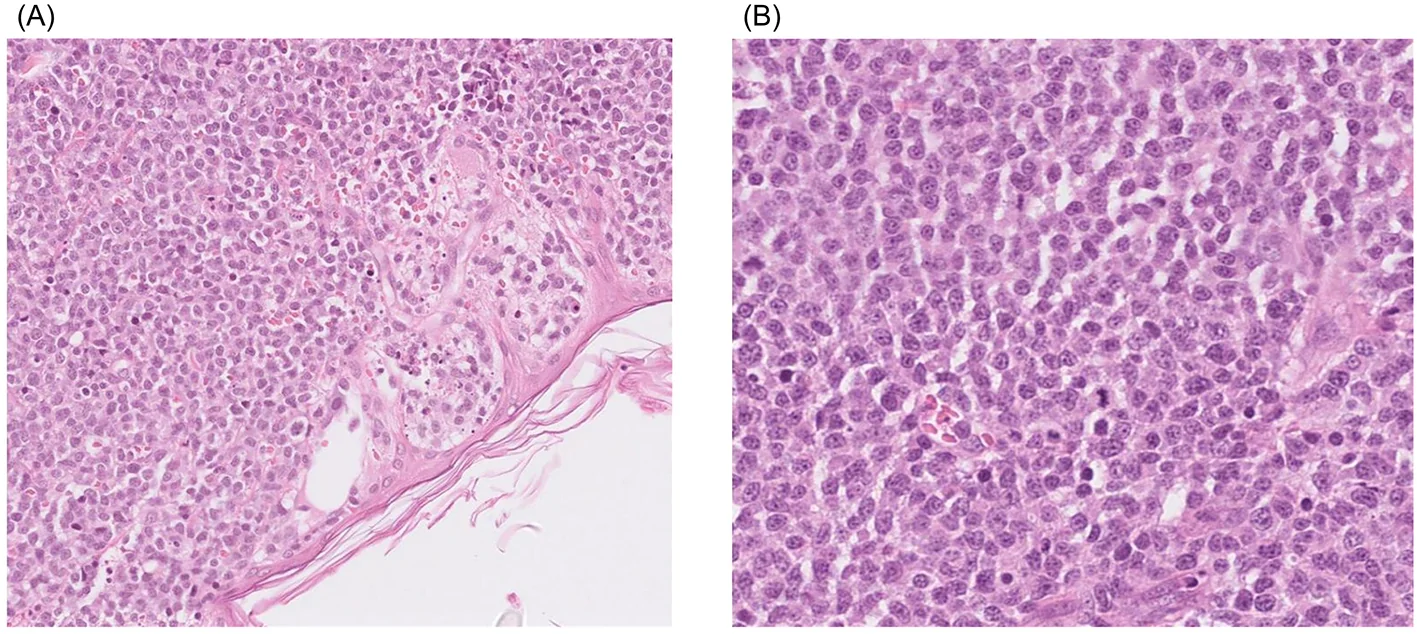
Histopathologic features of the left flank cutaneous mass (hematoxylin and eosin stain). **(A)** Dense sheets of neoplastic round cells expand the superficial dermis and multifocally invade the surface epithelium, consistent with epitheliotropism. 20 × objective magnification. **(B)** At higher magnification, the neoplastic cells consist of intermediate to large lymphocytes with scant to moderate eosinophilic cytoplasm, round to ovoid nuclei, moderate anisocytosis and anisokaryosis, and occasional mitotic figures. 40 × objective magnification.

### 2.4 Disease progression, rescue therapy, and clinical outcome

One week later (week 48), swelling of the right lower conjunctiva was noted (Figure 1E). Ocular ultrasonography revealed a 4.5-mm hypoechoic lesion involving the right lower eyelid and ventrolateral periocular region, suggestive of ocular lymphoma involvement based on the clinical history. Because the disease continued to progress during the second cycle of the reinitiated L-CHOP protocol, treatment was transitioned to rescue protocols. Sequential rescue therapies included a combination of L-asp, lomustine, and prednisolone; lomustine, vincristine, procarbazine, and prednisolone; and finally, continuous L-asp administration. Despite these treatments, the existing cutaneous lesions continued to enlarge, and new cutaneous masses developed at various locations throughout the body.

At week 58, a second palliative ECT session was performed using the same protocol as previously described, targeting the largest lesions on the upper and lower lips, inguinal region, and perineal region. Two days after ECT, a slight decrease in the size of the treated cutaneous masses was noted on gross examination and palpation. At reassessment 2 days later, no further reduction was observed, whereas the untreated cutaneous lesions continued to enlarge. The dog subsequently became increasingly lethargic and anorexic. Four days after the second ECT session, the dog developed respiratory distress, and thoracic radiographs revealed bilateral caudal interstitial pulmonary infiltrates. Despite supportive care, the dog showed progressive clinical deterioration, and the owner opted for euthanasia at week 59.

### 2.5 Postmortem examination

With the owner's consent, postmortem tissue samples were obtained from the right submandibular lymph node, cutaneous masses in the inguinal and left popliteal regions, and both caudal lung lobes. The right submandibular lymph node was effaced by sheets of large neoplastic lymphocytes, indicating diffuse large cell lymphoma. The cutaneous masses showed similar neoplastic lymphoid infiltration with multifocal invasion of the surface epithelium, consistent with epitheliotropism (Figure 3). Additional IHC confirmed DLBCL in both the lymph node and cutaneous lesions, in agreement with the findings in the previously examined skin masses. In contrast, both caudal lung lobes showed subacute interstitial pneumonia with alveolar septal inflammation, intra-alveolar fibrin and macrophages, and type II pneumocyte hyperplasia, without evidence of neoplastic infiltration.

**Figure 3:**
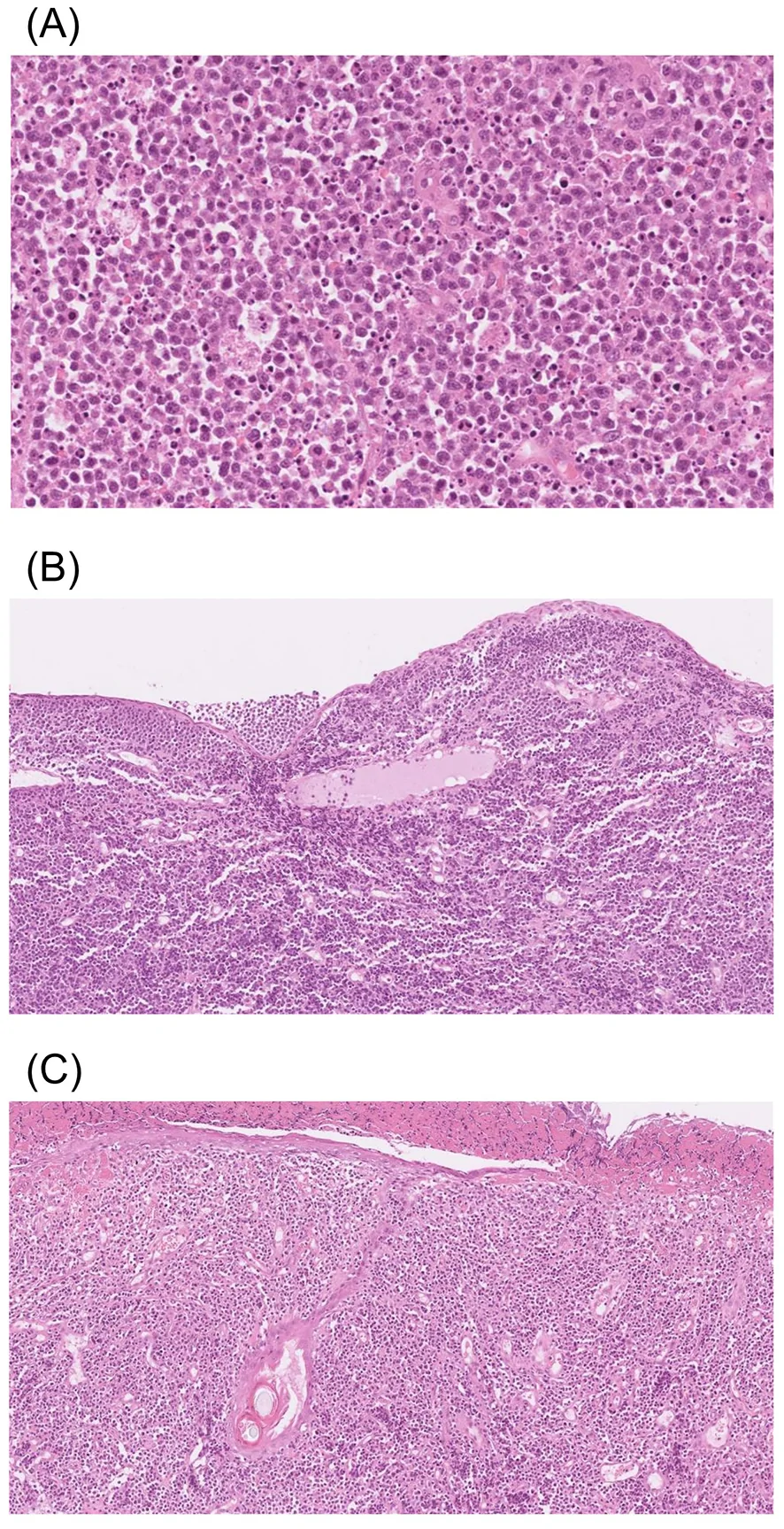
Histopathologic features of the right submandibular lymph node and cutaneous lesions obtained at postmortem examination. (Hematoxylin and eosin stain. Objective magnifications: A, 20 ×; B and C, 10 ×). **(A)** The right submandibular lymph node is effaced by dense sheets of large neoplastic lymphocytes with scattered tingible body macrophages, consistent with diffuse large cell lymphoma. **(B, C)** Cutaneous masses from the inguinal region and left hindlimb show infiltrative sheets and rows of neoplastic lymphocytes expanding the subepithelial connective tissue and multifocally invading the surface epithelium, consistent with epitheliotropism. Immunohistochemistry, not shown, confirmed diffuse CD20 expression in neoplastic cells from all three sites, with CD3 labeling limited to scattered small background lymphocytes.

## 3 Discussion

The dog described in this report was initially diagnosed with multicentric lymphoma and subsequently developed multiple cutaneous masses during the disease course. The lymph node and cutaneous lesions shared similar histological characteristics, and IHC confirmed DLBCL in both sites. These findings support the interpretation that the skin lesions represented secondary cutaneous involvement of the previously diagnosed lymphoma rather than an independent primary cutaneous neoplasm.

Epitheliotropism is most commonly associated with cutaneous T-cell lymphoma (CTCL) in dogs, particularly mycosis fungoides (3). Histologically, canine CTCL is characterized by infiltration of neoplastic lymphocytes into the epidermis and adnexal structures (4–6). Immunohistochemically, most cases express T-cell markers such as CD3, whereas B-cell markers, including CD20 and CD79a, are typically absent (7). In the present case, the cutaneous lesions showed histological features consistent with this pattern. However, in contrast to typical CTCL, the neoplastic cells showed diffuse CD20 expression with only scattered CD3-positive background lymphocytes in this case. These findings indicate that an epitheliotropic pattern, although highly suggestive of CTCL, is not exclusive to T-cell lineage.

Rare cases of epitheliotropic B-cell lymphomas have been reported in human medicine. These include both primary cutaneous B-cell lymphomas, most commonly marginal zone lymphoma (8, 9, 11) and secondary cutaneous involvement by systemic B-cell lymphomas (12, 13). In such cases, the skin lesions often showed dermal lymphoid infiltrate with epidermal invasion, but immunohistochemistry confirmed a B-cell phenotype with expression of CD20 and/or CD79a. Epitheliotropism has also been reported in an Epstein-Barr virus-positive DLBCL, in which CD20-positive B lymphocytes were identified within the epidermis (14). Although this pattern has not been previously described in canine B-cell lymphoma, this case suggests that DLBCL in dogs may also show a histological pattern resembling epitheliotropic T-cell lymphoma.

The clinical significance of secondary cutaneous involvement by DLBCL has been evaluated in human patients. In that study, secondary cutaneous DLBCL was associated with more advanced disease and poorer outcome than primary cutaneous DLBCL of the leg type (12). In the present case, multiple cutaneous lesions developed despite ongoing chemotherapy, even during periods when peripheral lymphadenopathy achieved temporary remission. This clinical course suggests that secondary cutaneous involvement of multicentric DLBCL may represent a more aggressive and treatment-resistant disease manifestation.

In addition to its diagnostic significance, this case illustrates the challenge of managing progressive cutaneous involvement of DLBCL. ECT was performed at two stages of the disease course for local control of cutaneous lesions. The first ECT session was selected because the lip mass developed near the completion of the UW-25 protocol while the peripheral lymph nodes remained in complete remission, and the lesion caused pain and difficulty with food intake. This treatment resulted in marked reduction of the lip mass to a non-palpable size within one month. In contrast, the response to the second ECT session was more limited and could not be definitively assessed because of the short follow-up. Although systemic chemotherapy remains the mainstay of treatment for canine lymphoma, ECT may have potential as a local palliative approach for solitary or symptomatic cutaneous lymphoma lesions causing pain, discomfort, or functional impairment (15). These clinical observations in this case should be interpreted cautiously, and further investigation is needed to determine the clinical value of ECT in this setting.

Near the end of the disease course, the dog developed respiratory distress with bilateral pulmonary infiltrates, and postmortem examination revealed interstitial pneumonia. Bleomycin-associated pulmonary toxicity was considered a possible cause because experimentally induced lesions in dogs show overlapping histologic features, including type II pneumocyte hyperplasia and mixed interstitial and alveolar inflammatory changes (16). However, the time interval between bleomycin administration and the onset of pulmonary infiltrates, as well as the histologic pattern, differed from those described in the experimental study. Other causes, including sepsis, infection, and adverse reactions to other drugs, could not be excluded; therefore, the etiology of the interstitial pneumonia remained undetermined.

A limitation of this report is that clonality testing was not performed to confirm a common origin of the nodal and cutaneous lesions. Nevertheless, their concordant histologic morphology, immunophenotype, and clinical course favored secondary cutaneous involvement by the previously diagnosed DLBCL.

## 4 Conclusion

To the best of the authors' knowledge, this is the first report of canine multicentric DLBCL that subsequently developed epitheliotropic cutaneous lesions most consistent with secondary involvement. This case indicates that, in dogs with pre-existing multicentric lymphoma, cutaneous lesions showing epidermal invasion should not be classified as T-cell lymphoma based on histological appearance alone. IHC is therefore essential for accurate immunophenotypic classification of such lesions. In addition, ECT may be considered as a palliative treatment option for selected cutaneous lymphoma lesions.

## Data Availability

The raw data supporting the conclusions of this article will be made available by the authors, without undue reservation.
